# Fauna Europaea: Hymenoptera – Symphyta & Ichneumonoidea

**DOI:** 10.3897/BDJ.5.e14650

**Published:** 2017-07-27

**Authors:** Kees van Achterberg, Andreas Taeger, Stephan M. Blank, Kees Zwakhals, Matti Viitasaari, Dicky Sick Ki Yu, Yde de Jong

**Affiliations:** 1 Naturalis, Leiden, Netherlands; 2 Senckenberg Deutsches Entomologisches Institut, Müncheberg, Germany; 3 Dr. Dreeslaan 204, Arkel, Netherlands; 4 Unaffiliated, Helsinki, Finland; 5 Unaffiliated, Ontario, Canada; 6 University of Amsterdam - Faculty of Science, Amsterdam, Netherlands; 7 Natural History Museum, Rotterdam, Rotterdam, Netherlands; 8 Museum für Naturkunde, Berlin, Germany

**Keywords:** Biodiversity, Informatics, Fauna Europaea, Taxonomic indexing, Zoology, Hymenoptera, Symphyta, Ichneumonoidea, Fauna Europaea, Taxonomic indexing

## Abstract

*Fauna Europaea* provides a public web-service with an index of scientific names (including important synonyms) of all extant European terrestrial and freshwater animals, their geographical distribution at the level of countries and major islands (west of the Urals and excluding the Caucasus region), and some additional information. The *Fauna Europaea* project comprises about 230,000 taxonomic names, including 130,000 accepted species and 14,000 accepted subspecies, which is much more than the originally projected number of 100,000 species. *Fauna Europaea* represents a huge effort by more than 400 contributing specialists throughout Europe and is a unique (standard) reference suitable for many users in science, government, industry, nature conservation and education.

For the Hymenoptera, taxonomic data from one grade (Symphyta) and one Superfamily (Ichneumonoidea), including 15 families and 10,717 species, are included.

Ichneumonoidea is the largest superfamily of Hymenoptera and consisting of two extant families, Ichneumonidae and Braconidae. The costal cell of the fore wing is absent, the fore wing has at least two closed cells, the constriction between the mesosoma (thorax + first abdominal segment or propodeum) and the metasoma (remainder of abdomen) is distinct and the parasitoid larvae usually spin a silken cocoon. Also, the metasoma is ventrally partly desclerotized in the vast majority of ichneumonoids.

## Introduction

In 1998 the European Commission published the European Community Biodiversity Strategy, providing a framework for development of Community policies and instruments in order to comply with the Convention on Biological Diversity. This Strategy recognises the current incomplete state of knowledge at all levels concerning biodiversity, which is a constraint on the successful implementation of the Convention. *Fauna Europaea* contributes to this Strategy by supporting one of the main themes: to identify and catalogue the components of European biodiversity into a database to serve as a basic tool for science and conservation policies.

With regard to biodiversity in Europe, science and policies depend on sufficient knowledge of its components. The assessment of biodiversity, monitoring changes, sustainable exploitation of biodiversity, as well as much legislative work depends upon a validated taxonomic overview, in which *Fauna Europaea* plays a major role by providing a web-based information infrastructure with an index of scientific names (including important synonyms) of all living European land and freshwater animals, their geographical distribution at the level of countries and some relevant optional information. In this sense the *Fauna Europaea* database provides a unique reference for many user-groups such as scientists, governments, industries, conservation communities and educational programs.

*Fauna Europaea* (FaEu) kicked off in 2000 as an EC-FP5 four-year project, delivering its first release in 2004 (Jong de et al. 2014). *Fauna Europaea* has continuously been updated, and after a further decade of steady progress, to efficiently disseminate the results of *Fauna Europaea* and to properly credit the *Fauna Europaea* contributors, modern e-publishing tools are being applied to prepare data papers on all 58 major taxonomic groups. For this purpose a special Biodiversity Data Journal Series has been compiled, called Contributions on Fauna Europaea (see also: Pensoft News item 17 Dec 2014). This work was initiated during the ViBRANT project and is further supported by the recently started EU BON project.

In the EU BON project also further steps will be made to implement *Fauna Europaea* as a basic tool and standard reference for biodiversity research and as a means to facilitate taxonomic expertise evaluation and management in Europe, including its contributions to the European Taxonomic Backbone (PESI / EU-nomen) project (Jong de et al. 2015).

This paper is the first publication of the *Fauna Europaea* Hymenoptera (Symphyta & Ichneumonoidea) data sector as a BDJdata paper in the *Fauna Europaea* series.

Although practically treated as a grade, Symphyta form a paraphyletic assemblage and are not a monophyletic clade; the name is used for all Hymenoptera with the abdomen broadly connected to the thorax **and** having wings with several closed cells **or** with cenchri present. The fore tibia has often two apical spurs and the larvae are usually caterpillar-like (“false caterpillars”, with 6-8 pairs of abdominal legs). Larvae boring in wood or stems are legless grub-like larvae; ovipositor usually very wide and saw-like, but narrow in Siricidae, Xiphydriidae and Orussidae.

## General description

### Purpose

*Fauna Europaea* is a database of the scientific names and distributions (at national or in some cases regional level) of all currently known extant multicellular European terrestrial and freshwater animal species. The database has been assembled by a large network of taxonomic specialists. An extended description of the *Fauna Europaea* project can be found in Jong de et al. 2014. A summary is given in the sections below.

**Figure 1.**
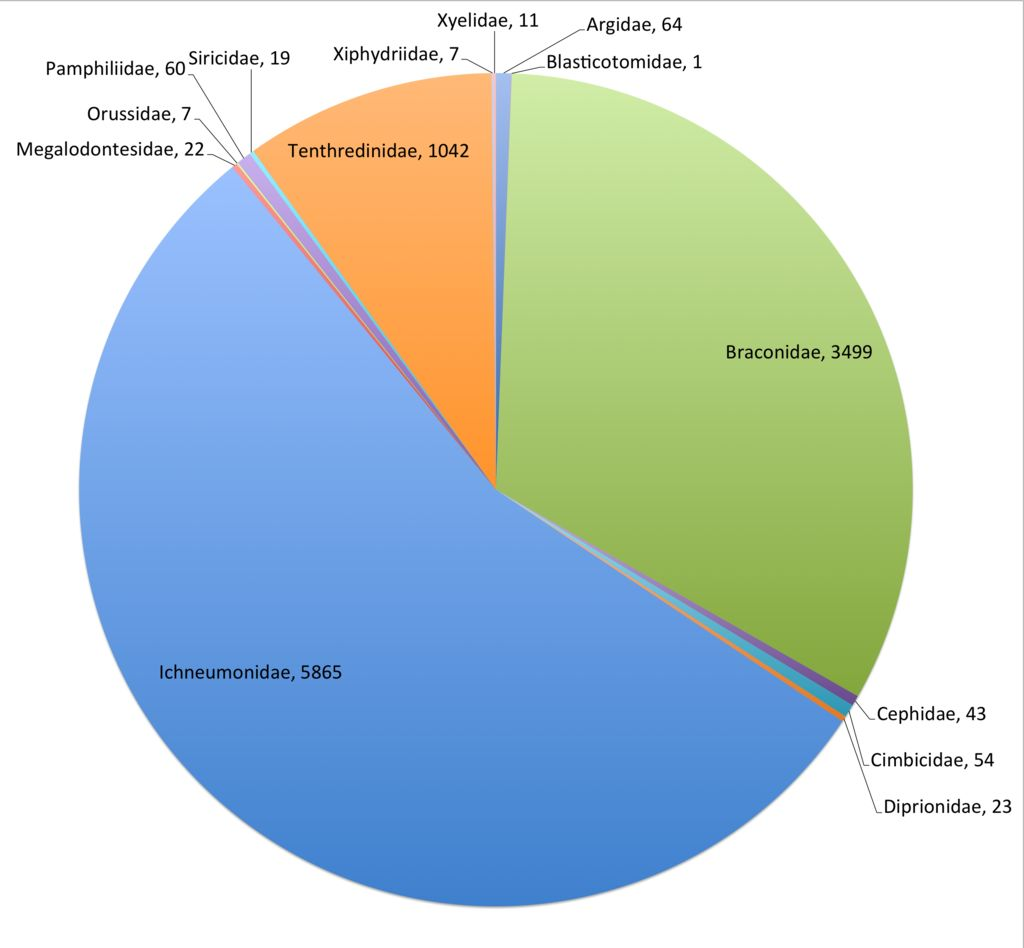
FaEu Hymenoptera (Symphyta & Ichneumonoidea) species per family. See Table 1 for family statistics.

The Hymenoptera (Symphyta & Ichneumonidae) is one of the 58 Fauna Europaea major taxonomic groups, covering **10,717** species (Fig. 1). The data were collated by a network of 7 specialists (Table 1,Table 2).

### Additional information

Introduction Hymenoptera (Symphyta & Ichneumonoidea)

**Figure 2.**
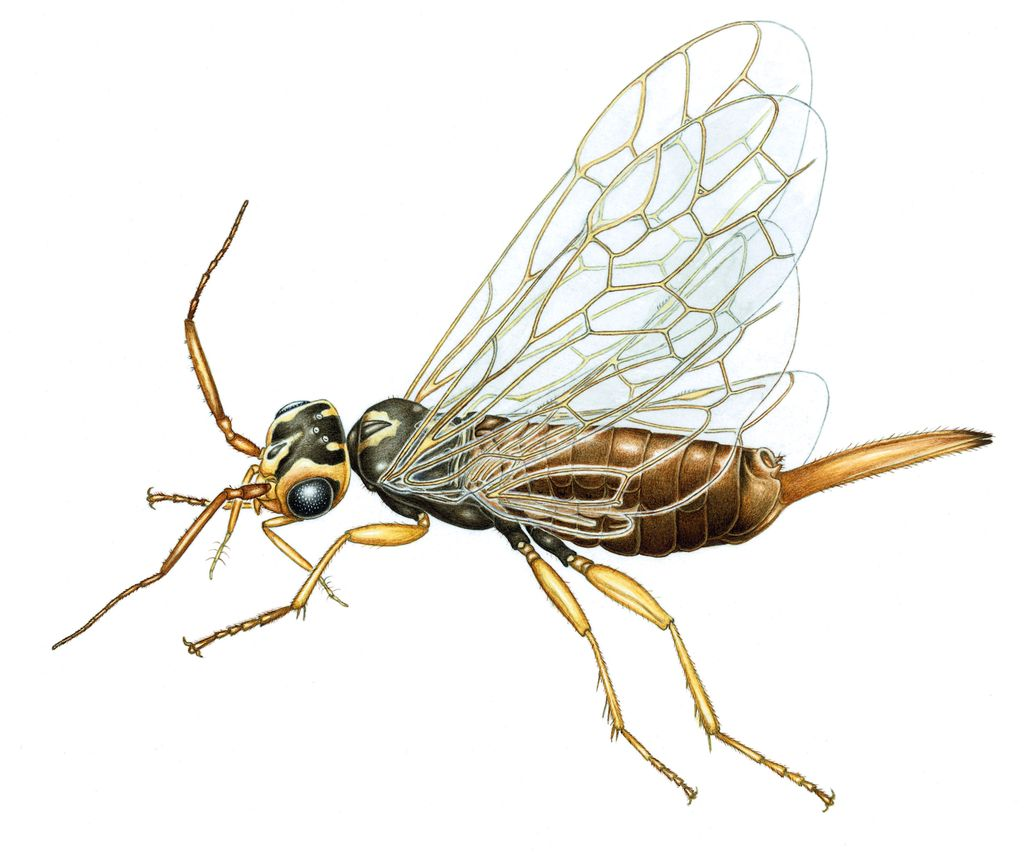
Xyelidae *Xyela julii* (Brebisson, 1818). Drawing by Erik-Jan Bosch.

The Symphyta contain the following 13 families in Europe:

Xyelidae: Oldest extant family of Hymenoptera with small number of extant species in the Holarctic region. The imagines visit flowering (birch-)catkins, near the conifers where the larvae eat pollen in the cones of pines (*Pinus* spp.) and new buds and shoots of firs (*Abies* spp.). The maxillary palpi are aberrant and used as a grasping instrument (Fig. 2).

Megalodontesidae: Small family formerly known as Megalodontidae and occurring in the subtropical parts of the Palaearctic region. Most larvae live (solitarily) in a case, which is generally suspended among or on the leaves of umbelliferous plants, on which the larvae feed.

**Figure 3.**
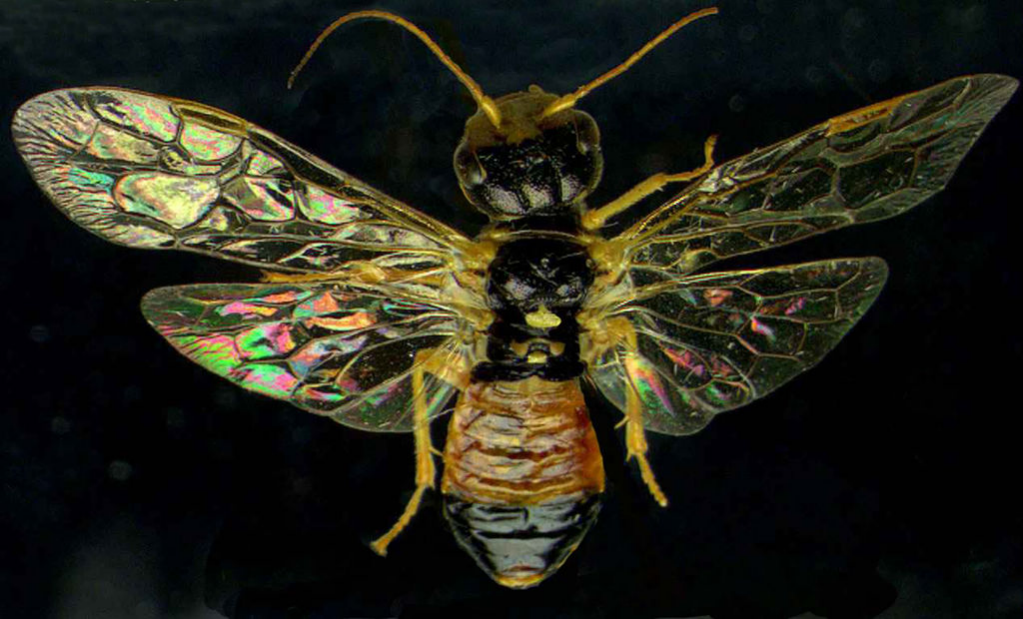
*Pamphilius aurantiacus* (Giraud, 1857). Photo by C. van Achterberg.

Pamphiliidae: Medium-sized family occurring in the Holarctic region. The larvae generally live solitarily in spun cases or curled-up leaves of Rosaceae and other deciduous plants or gregariously on coniferous trees (Fig. 3), although some Neurotoma (on Rosaceae) are gregarious.

**Figure 4.**
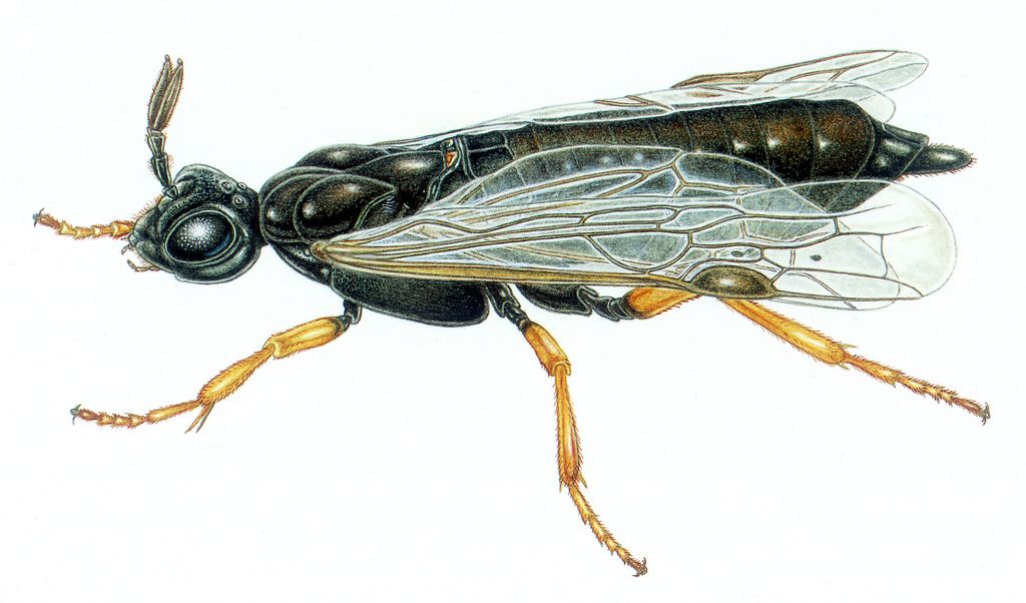
*Blasticotoma* Klug, 1834. Drawing by Erik-Jan Bosch.

Blasticotomidae: A very small family (with only two genera and 12 extant species) and now confined to temperate forests of the Palaearctic region. The larva of the only European species, *Blasticotoma filiceti* Klug, 1834, lives in the stems of ferns in a small chamber (or gall) and feed on the phloem fluids (Fig. 4).

Argidae: Medium -sized cosmopolitan family; the larvae of Arginae live especially on Rosaceae, but also catkin-bearing trees (Salicaceae, Fagaceae) and Proteaceae are used. Larvae of some Sterictiphorinae live on herbaceous Fabaceae. The males in the subfamily Sterictiphorinae may have the third antennal segment branched.

**Figure 5.**
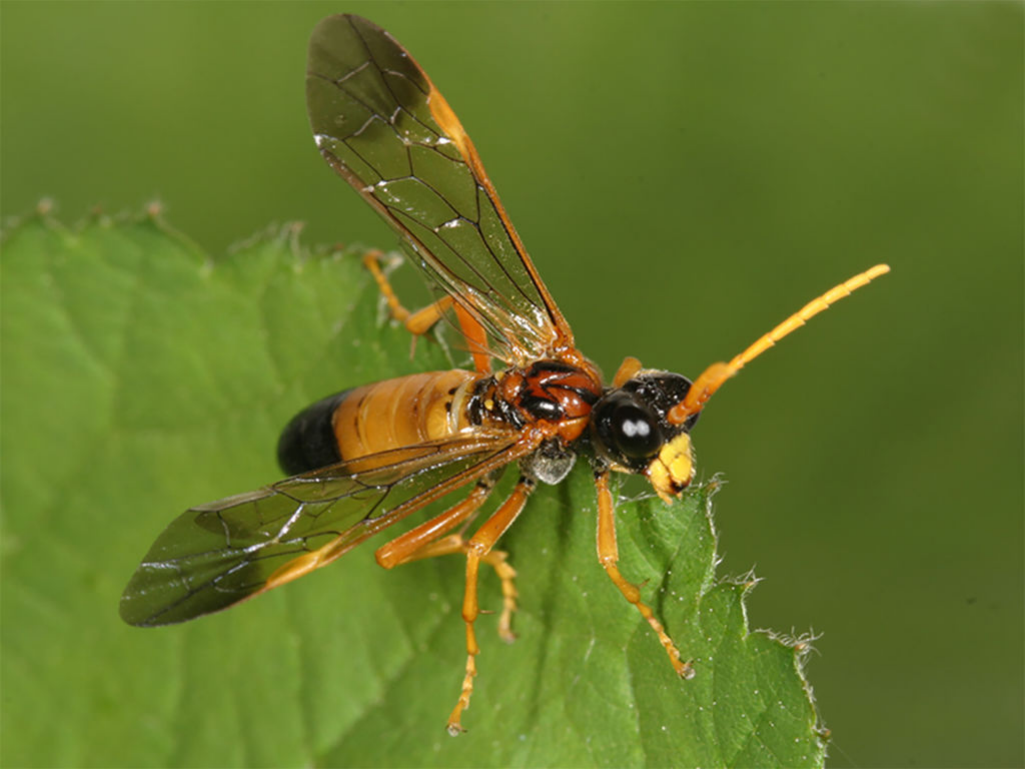
Tenthredinidae *Tenthredo campestris* Linnaeus, 1758. Photo by H. Berkhoudt.

**Figure 6.**
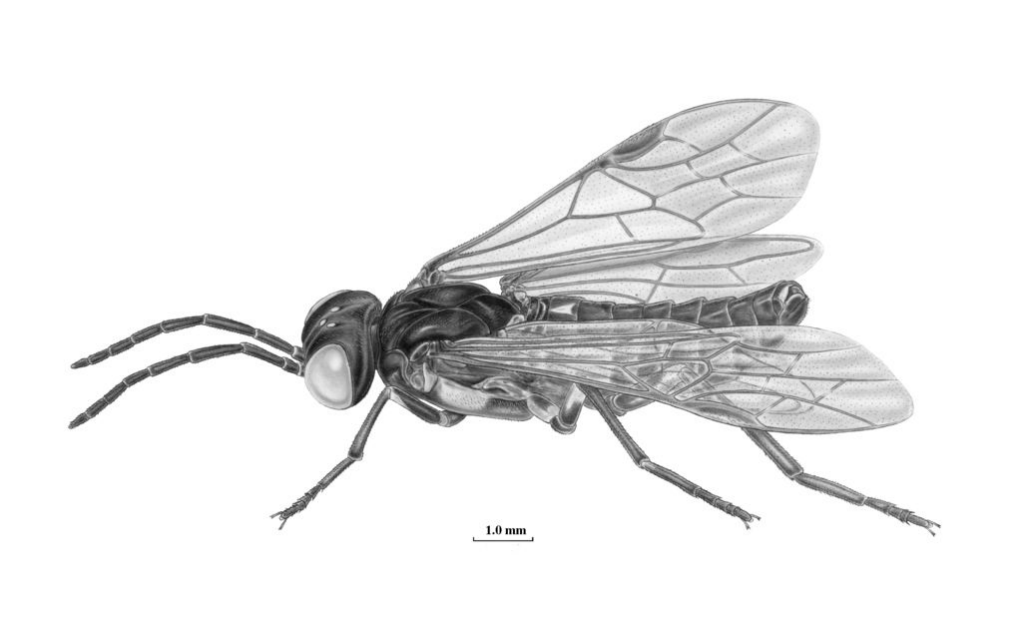
Tenthredinidae (Tenthredininae) Latreille, 1802. Drawing by Bas Blankevoort.

Tenthredinidae: Large and very diverse cosmopolitan family containing most species of Symphyta in the Holarctic region. The larvae live on or mine on many kinds of trees, shrubs or herbaceous plants, including fruits, ferns, horse-tails (*Equisetum* spp.) and rushes (*Juncus* species) (Fig. 5, Fig. 6).

Heptamelidae: Small Old World family with only two genera and 36 extant species. The European Heptamelus species use ferns as their larval food: the egg is entirely inserted in the rachis or petiole of the fern and the larva bores in the petiole of fern. Previously treated as a tribe of Tenthredinidae-Selandriinae, but raised to family rank by Malm & Nyman (Malm and Nyman 2014). Larva of Pseudoheptamelus is exophytic.

**Figure 7.**
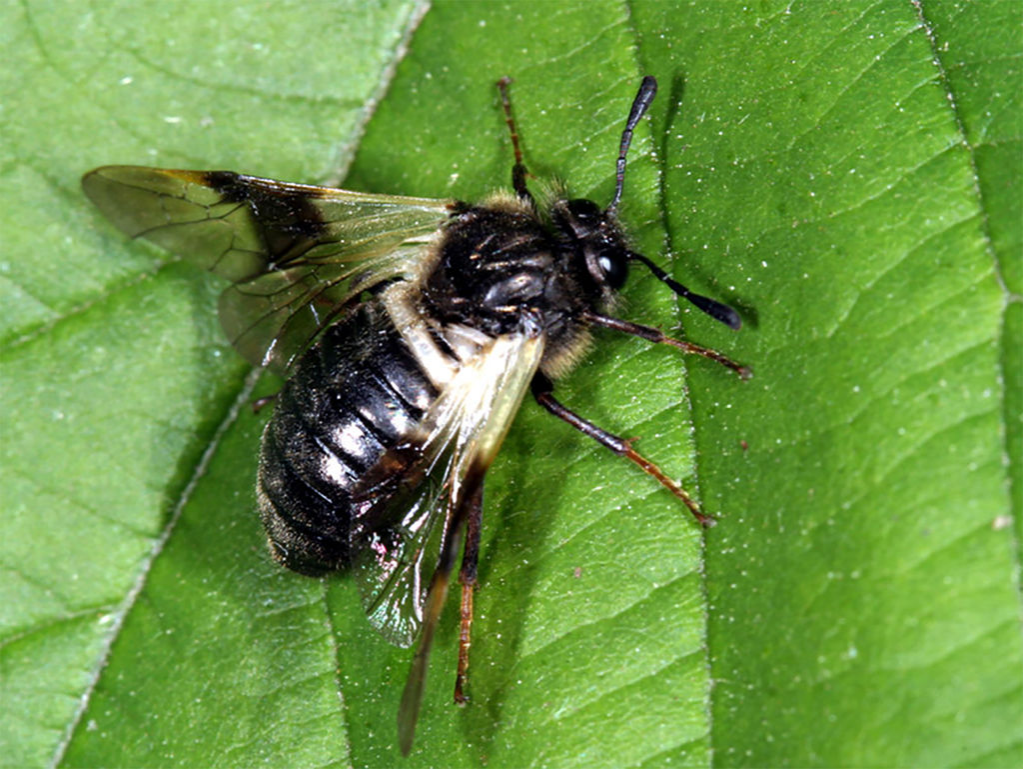
Cimbicidae *Abia fasciata* (Linnaeus, 1758). Photo by H. Berkhoudt.

Cimbicidae: Rather small Holarctic family. The often conspicuous larvae live on various deciduous trees, climbers (*Lonicera* spp.) and herbs (Fig. 7).

Diprionidae: Rather small and mainly Holarctic family. The larvae live (often gregariously) on conifers, and may become a serious pest. Adult males of several species have conspicuously branched antennae.

Cephidae: Rather small and mainly Holarctic family. The larvae bore in twigs of deciduous trees and shrubs or in stems of Gramineae (Poaceae).

**Figure 8.**
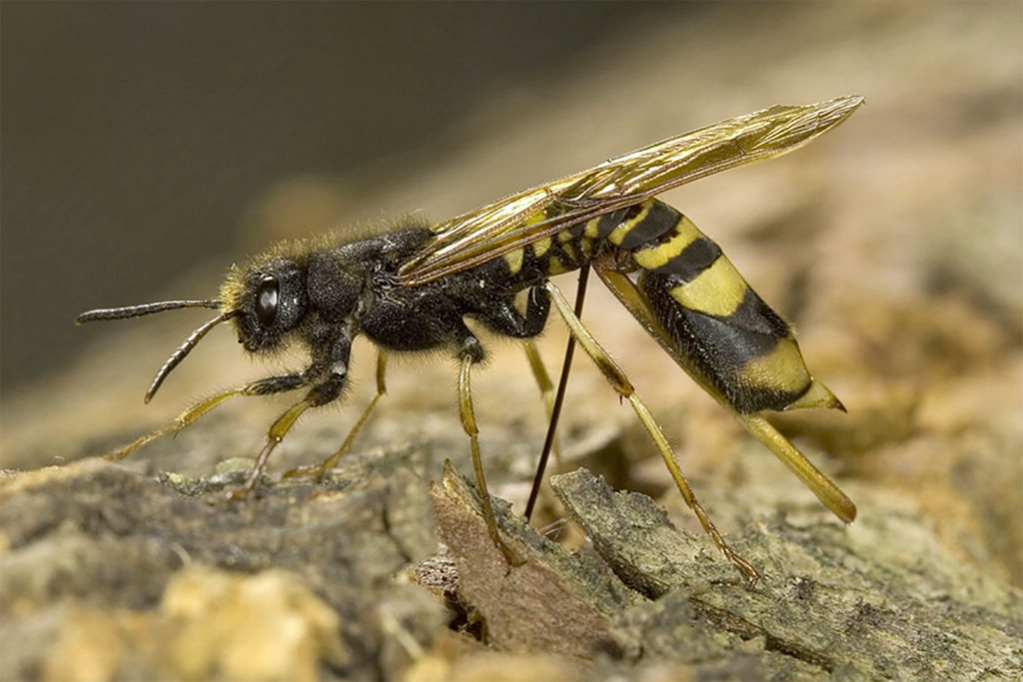
Siricidae *Tremex fuscornis* (Fabricius, 1787). Photo by R. Krekels.

Siricidae: Rather small and mainly Holarctic family, but introduced with timber into other areas. The larvae bore in wood of coniferous and deciduous trees and shrubs, stems of Gramineae (Poaceae), or sometimes in herbs." (e.g. Pachycephus in Papaver). Adults have their wings more or less longitudinally wrinkled (Fig. 8).

**Figure 9.**
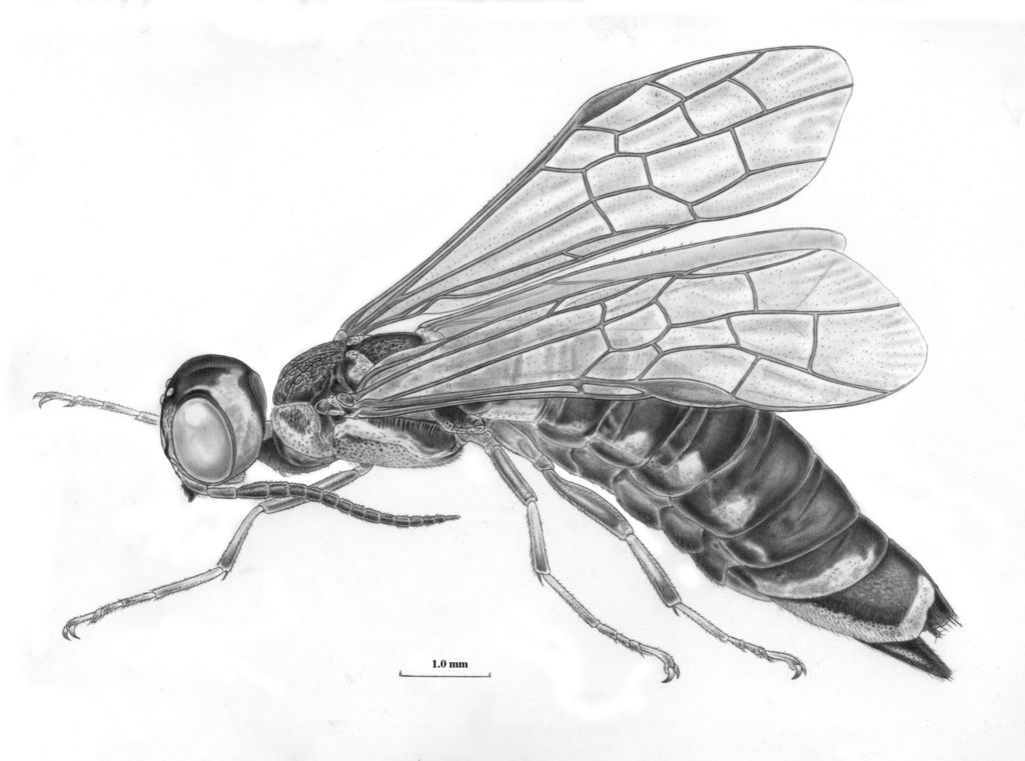
Xiphydriiidae. Drawing by Bas Blankevoort.

Xiphydriidae: Rather small family, known from all regions except the Afrotropical region. The eggs are laid into smaller limbs and branches of deciduous trees wherein the larvae make their burrows (Fig. 9).

**Figure 10.**
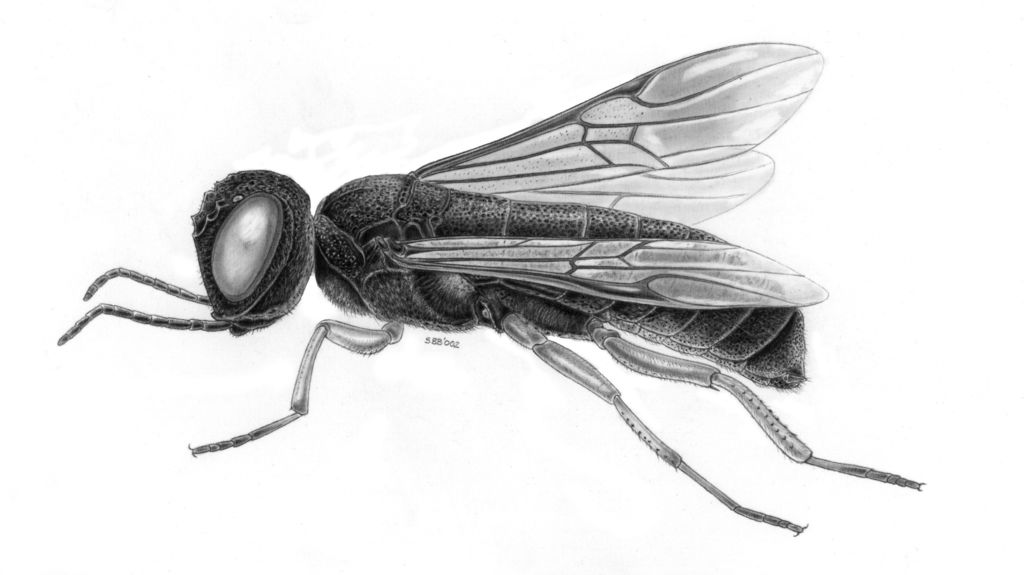
Orussidae Latreille, 1797. Drawing by Bas Blankevoort.

Orussidae: Small cosmopolitan family; the larvae are idiobiont parasitoids of wood inhabiting larvae: mainly of Buprestidae, but also of Curculionidae, Cerambycidae, Siricidae and (as hyperparasitoid) Ichneumonidae (Rhyssinae). The first instar larva is a solitary pseudohyperparasitoids, but subsequent instars may feed endophagously in the dead host. Only family of Symphyta with upper valve of ovipositor undivided apically Quicke et al. 1994 and has the long fine ovipositor internalised (Fig. 10).

Braconidae have the second and third tergites of metasoma immovably joined, vein 2m-cu of fore wing nearly always absent and vein 1r-m of hind wing more or less basally of distal end of vein SC+R or absent. Very large family, with about 21,300 valid species described. The biology is very variable (Shaw and Huddleston 1991); the legless larvae are ectoparasitoids of all kinds of larvae of insects, egg-larval endoparasitoids, true larval endoparasitoids of several orders of insects (but not as hyperparasitoids; only one genus contains pseudohyperparasitoids) to endoparasitoids of adult beetles, bugs and aphids; exceptionally of adult Apidae and parasitoid wasps. The Aphidiinae are a basal group of the Braconidae and in the past often treated as a separate family Aphidiidae, which is not justified by their phylogeny (Quicke and van Achterberg 1990).

Ichneumonidae is the largest family of the Hymenoptera (and one of the largest families of the animal kingdom) with about 25,300 valid species described. Ichneumonidae have the venation of the fore wing rather stable, in contrast to the highly variable venation of Braconidae. The second metasomal tergite is hinged to third tergite (but still movable and not immobilised as in Braconidae), vein 2m-cu of fore wing nearly always present and vein 1r-m of hind wing more or less distally of distal end of vein SC+R or absent. The larvae are parasitoids in or on all kinds of larvae of several orders of insects, but especially of caterpillars and also rather frequent (as pseudohyperparasitoids) of other parasitic Hymenoptera: especially the Cryptinae (the largest subfamily, another part frequently parasitizes egg aggregates of spiders!). The presence of a discosubmarginal cell is also characteristic (but does not exclude all Braconidae). Highly aberrant are the Holarctic Hybrizontinae (formerly included in the Braconidae and often known by its synonym Paxylommatinae) in morphology (e.g. wings) and biology (parasitoids of ant larvae).

## Project description

### Title

This BDJ data paper includes the taxonomic indexing efforts in *Fauna Europaea* on European Hymenoptera (Symphyta & Ichneumonoidea) covering the first two versions of *Fauna Europaea* worked on between 2000 and 2013 (up to version 2.6).

### Personnel

The taxonomic framework of *Fauna Europaea* includes partner institutes, which together with a number of additional experts, and local scientists provide the taxonomic expertise, taking care of data collation and faunistic quality assurance.

Every taxonomic group is covered by at least one Group Coordinator responsible for the supervision and integrated input of taxonomic and occurrence data of a particular group. For Hymenoptera (Symphyta & Ichneumonoidea) the responsible Group Coordinator is Kees van Achterberg.

The *Fauna Europaea* checklist would not have reached its current level of completion without the input from several groups of specialists. The formal responsibility of collating and delivering the data of relevant families has resided with the below appointed Taxonomic Specialists (see Table 1), while Associate Specialists deserve credit for their important contributions at various levels, including particular geographic regions or (across) taxonomic groups (see Table 2).

Data management tasks were taken care about by the *Fauna Europaea* project bureau. During the project phase (until 2004) a network of principal partners took care about diverse management tasks: Zoological Museum Amsterdam (general management & system development), Zoological Museum of Copenhagen (data collation), National Museum of Natural History in Paris (data validation) and Museum and Institute of Zoology in Warsaw (Newly Associated States [NAS] extension). From the formal termination of the project in 2004 to 2013, all tasks were taken over by the Zoological Museum Amsterdam.

### Study area description

**Figure 11.**
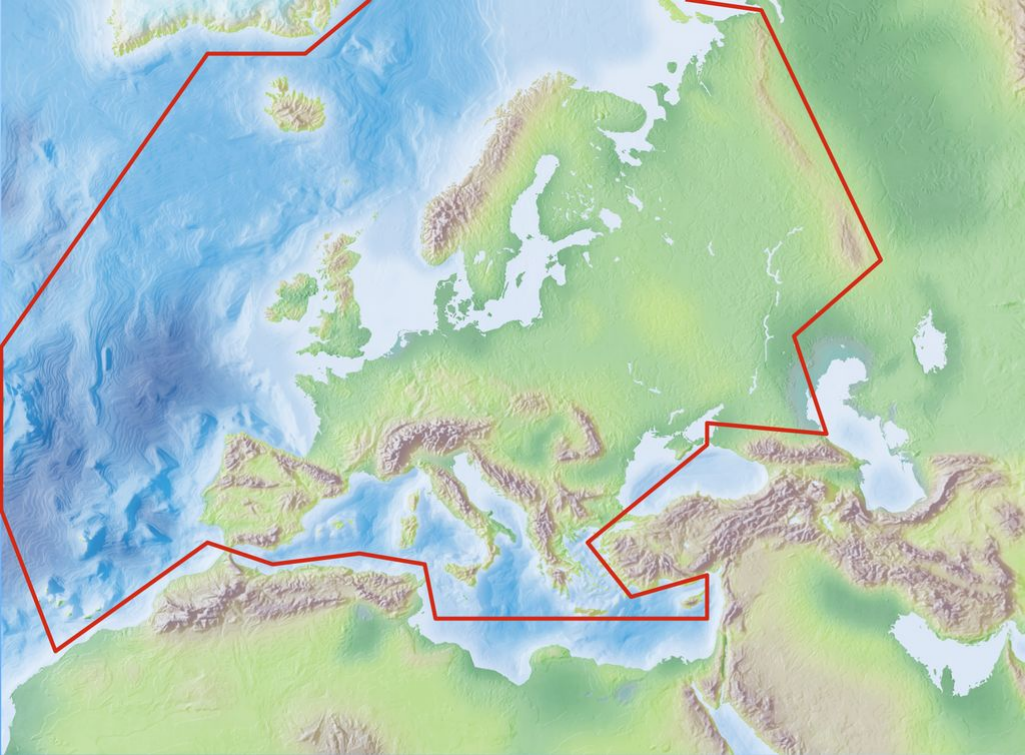
Fauna Europaea geographic coverage ('minimal Europe').

The area study covers the European mainland (Western Palaearctic), including the Macaronesian islands, excluding the Caucasus, Turkey, Arabian Peninsula and Northern Africa (Fig. 11).

### Design description

*Standards*. Group coordinators and taxonomic specialists have been delivering the (sub)species names according to strict standards. The names provided by FaEu are scientific names. The taxonomic scope includes issues like, (1) the definition of criteria used to identify the accepted species-group taxa, (2) the hierarchy (classification scheme) for the accommodation of the all accepted species and (3), relevant synonyms, and (4) the correct nomenclature. The 'Fauna Europaea Guidelines for Group Coordinators and Taxonomic Specialists' Suppl. material 2, include the standards, protocols, scope, and limits that provide the instructions for all more than 400 specialists contributing to the project.

**Figure 12.**
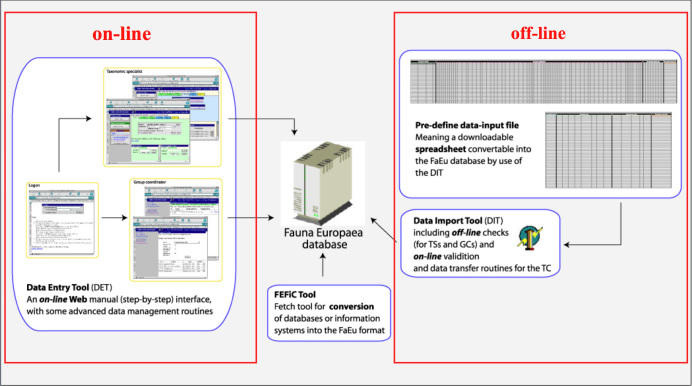
Fauna Europaea on-line (browser interfaces) and off-line (spreadsheets) data entry tools.

*Data management*. The data records could either be entered off-line into a pre-formatted MS-Excel worksheet or directly into the Fauna Europaea transaction database using an on-line browser interface (Fig. 12). The data servers were hosted at the Academic Informatics Center of the University of Amsterdam (SARA/Vancis). Since 2013 the data servers are hosted at the Museum für Naturkunde in Berlin, and a new data entry (update) tool is under development.

*Data set*. The *Fauna Europaea* basic data set consists of: accepted (sub)species names (including authorship), synonyms (including authorship), taxonomic hierarchy / classification, misapplied names (including misspellings and alternative taxonomic views), homonym annotations, expert details, European distribution (at country level), Global distribution (only for European species), taxonomic reference (optional), occurrence reference (optional).

### Funding

*Fauna Europae*a was funded by the European Commission under the Fifth Framework Programme and contributed to the Support for Research Infrastructures work programme with Thematic Priority Biodiversity (EVR1-1999-20001) for a period of four years (1 March 2000 – 1 March 2004), including a short 'NAS extension', allowing EU candidate accession countries to participate. Follow-up support was given by the EC-FP5 EuroCAT project (EVR1-CT-2002-20011), by the EC-FP6 ENBI project (EVK2-CT-2002-20020), by the EC-FP6 EDIT project (GCE 018340), by the EC-FP7 PESI project (RI-223806) and by the EC-FP7 ViBRANT project (RI-261532). Continued management and hosting of the *Fauna Europaea* services was supported by the University of Amsterdam (Zoological Museum Amsterdam) and SARA/Vancis. Recently, the hosting of *Fauna Europaea* was taken over by the Museum für Naturkunde in Berlin, supported by the EC-FP7 EUBON project (grant agreement ENV-308454).

Additional support for preparing the Hymenoptera (Symphyta & Ichneumonoidea) data set was received through the numerous institutions allowing for the proper allocation of time by the contributing taxonomic specialists.

## Sampling methods

### Study extent

See spatial coverage and geographic coverage descriptions.

### Sampling description

*Fauna Europaea* data have been assembled by principal taxonomic experts, based on their individual expertise, including literature study, collection research, and field observations. In total no less than 476 experts contributed taxonomic and/or faunistic information for Fauna Europaea. The vast majority of the experts are from Europe (including EU non-member states). As a unique feature, F*auna Europaea* funds were set aside for paying/compensating for the work of taxonomic specialists and group coordinators (around five Euro per species).

To facilitate data transfer and data import, sophisticated on-line (web interfaces) and off-line (spreadsheets) data-entry routines have been built, well integrated within an underlying central Fauna Europaea transaction database (see Fig. 12). This includes advanced batch data import routines and utilities to display and monitor the data processing within the system. In retrospect, it seems that the off-line submission of data was probably the best for bulk import during the project phase, while the on-line tool was preferred to enter modifications in later versions. This system was operational until 2013 when dismantled for replacement to MfN.

**Figure 13.**
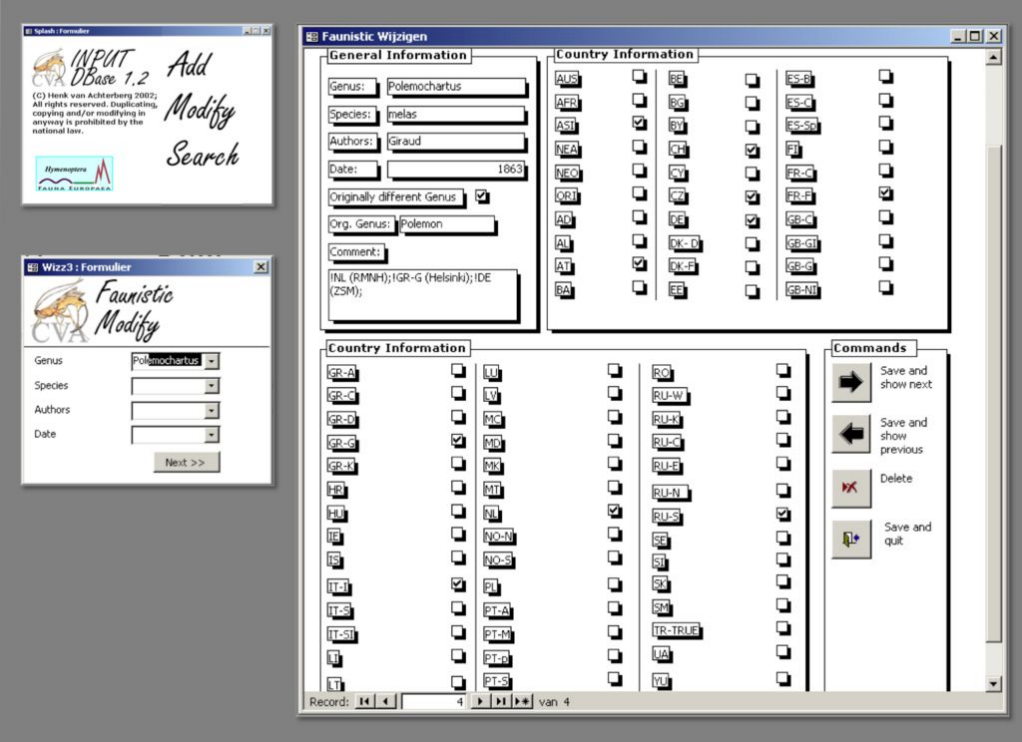
Fauna Europaea - Hymenoptera Visual Basic data import tool, showing interfaces for editing taxonomic and faunistic data.

For accumulating the data a Visual Basic tool was developed by Henk van Achterberg, including an export routine converting the underlying MS Access database into a suitable data import format (Fig. 13). This tool is still used for updating the database and is available for general use in other Fauna Europaea groups as well.

A first release of the Fauna Europaea index via the web-portal has been presented at 27^th^ of September 2004, the most recent release (version 2.6.2) was launched at 29 August 2013. A latest addition to the Hymenoptera (Symphyta & Ichneumonoidea) data was prepared for version 2.1 (December 2009).

### Quality control

*Fauna Europaea* data are unique in a sense that they are fully expert based. Selecting leading experts for all groups included a principal assurance of the systematic reliability and consistency of the Fauna Europaea data.

Furthermore, all *Fauna Europaea* data sets are intensively reviewed at regional and thematic validation meetings, at review sessions on taxonomic symposia (for some groups), by *Fauna Europaea* Focal Points (during the FaEu-NAS and PESI projects) and by various end-users sending annotations using the web form at the web-portal. Additional validation on gaps and correct spelling was effected at the validation office in Paris.

Checks on technical and logical correctness of the data were implemented by the data entry tools, including around 50 'Taxonomic Integrity Rules'. This validation tool proved to be of considerable value for both the taxonomic specialists and project management, and significantly contributed to the preparation of a remarkably clean and consistent data set.

This thorough review procedure makes *Fauna Europaea* the most scrutinised data set in its domain. In general we expected to get taxonomic data for 99.3% of the known European fauna directly after the initial release of *Fauna Europaea* (Jong de et al. 2014). The faunistic coverage is not quite as good, but is nevertheless 90-95% of the total fauna. Recognised gaps in Hymenoptera (Symphyta & Ichneumonoidea) are in major subfamilies of the Braconidae (e.g., small Opiinae, Alysiinae, Microgastrinae) and Ichneumonidae (especially Cryptinae and Campopleginae) (see Table 1).

To optimise the use and implementation of a uniform and correct nomenclature, a cross-referencing of the Fauna Europaea Hymenoptera (Symphyta & Ichneumonoidea) data-set with relevant taxonomic resources is recommended, also supporting the global efforts on establishing a global taxonomic resolution service, provisionally called 'Global Names Architecture' (Pyle and Michel 2008, Jong de et al. 2014). Applicable nomenclature databases specifically dedicated to these taxonomic groups include: the world catalogue on Ichneumonoidea: Taxapad (Yu et al. 2016) and the *Electronic World Catalog of Symphyta*: ECatSym (Taeger et al. 2010, Taeger et al. 2011). As a preparation, a semi-automatic validation on the *Taxapad* dataset has been carried out, using the PESI Taxon Match Tool and LifeWatch Backbone services. The result is used to annotate discrepancies between both *Taxapad* and *Fauna Europaea* and to mark gaps in the current version of *Fauna Europaea*, supporting data validation (Suppl. material 1).

### Step description

By evaluating team structure and life cycle procedures (data-entry, validation, updating, etc.), clear definitions of roles of users and user-groups, according to the taxonomic framework were established, including ownership and read and write privileges, and their changes during the project life-cycle. In addition, guidelines on common data exchange formats and codes have been issued (see also Suppl. material 2).

## Geographic coverage

### Description

Species and subspecies distributions in Fauna Europaea are registered at least a country level, meaning political countries. For this purpose the FaEu geographical system basically follows the TDWG standards. The covered area includes the European mainland (Western Palaearctic), plus the Macaronesian islands (excl. Cape Verde Islands), Cyprus, Franz Josef Land and Novaya Zemlya. Western Kazakhstan and the Caucasus are excluded (see Fig. 11).

The focus is on species (or subspecies) of European multicellular animals of terrestrial and freshwater environments. Species in brackish waters, occupying the marine/freshwater or marine/terrestrial transition zones, are generally excluded.

### Coordinates

Mediterranean and Arctic Latitude; Atlantic Ocean (Azores) and Ural Longitude.

## Taxonomic coverage

### Description

The Fauna Europaea database contains the scientific names of all living European land and freshwater animal species, including numerous infra-groups and synonyms. More details about the conceptual background of Fauna Europaea and standards followed are described in the project description papers.

This data paper covers the Hymenoptera (Symphyta & Ichneumonoidea) content of Fauna Europaea, including 14 Families 10,717 species, 39 subspecies and 761 (sub)species synonyms (see Fig. 1).

### Taxa included

**Table:** 

Rank	Scientific Name	
kingdom	Animalia	
subkingdom	Eumetazoa	
phylum	Arthropoda	
subphylum	Hexapoda	
class	Insecta	
order	Hymenoptera	
suborder	Apocrita	
superfamily	Ichneumonoidea	
family	Braconidae	
subfamily	Adeliinae (now: tribe Adeliini of subfamily Cheloninae)	
subfamily	Agathidinae	
subfamily	Alysiinae	
subfamily	Aphidiinae	
subfamily	Blacinae (now: tribe Blacini of subfamily Brachistinae)	
subfamily	Brachistinae	
subfamily	Braconinae	
subfamily	Cardiochilinae	
subfamily	Cenocoeliinae	
subfamily	Charmontinae	
subfamily	Cheloninae	
subfamily	Dirrhopinae	
subfamily	Doryctinae	
subfamily	Euphorinae	
subfamily	Exothecinae	
subfamily	Gnamptodontinae	
subfamily	Helconinae	
subfamily	Histeromerinae (now: tribe Histeromerini of subfamily Rhyssalinae)	
subfamily	Homolobinae	
subfamily	Hormiinae	
subfamily	Ichneutinae	
subfamily	Lysiterminae	
subfamily	Macrocentrinae	
subfamily	Microgastrinae	
subfamily	Microtypinae	
subfamily	Miracinae	
subfamily	Opiinae	
subfamily	Orgilinae	
subfamily	Pambolinae	
subfamily	Rhysipolinae	
subfamily	Rhyssalinae	
subfamily	Rogadinae	
subfamily	Sigalphinae	
family	Ichneumonidae	
subfamily	Acaenitinae	
subfamily	Adelognathinae	
subfamily	Agriotypinae	
subfamily	Alomyinae	
tribe	Alomyini	
subfamily	Anomaloninae	
tribe	Anomalonini	
tribe	Gravenhorstiini	
subfamily	Banchinae	
tribe	Atrophini	
tribe	Banchini	
tribe	Glyptini	
subfamily	Brachycyrtinae	
subfamily	Campopleginae	
subfamily	Collyriinae	
subfamily	Cremastinae	
subfamily	Cryptinae	
tribe	Cryptini	
tribe	Hemigasterini	
tribe	Phygadeuontini	
subfamily	Ctenopelmatinae	
tribe	Ctenopelmatini	
tribe	Euryproctini	
tribe	Mesoleiini	
tribe	Olethrodotini	
tribe	Perilissini	
tribe	Pionini	
tribe	Scolobatini	
subfamily	Cylloceriinae	
subfamily	Diacritinae	
subfamily	Diplazontinae	
subfamily	Eucerotinae	
subfamily	Hybrizontinae	
subfamily	Ichneumoninae	
tribe	Clypeodromini	
tribe	Ctenocalini	
tribe	Eurylabini	
tribe	Goedartiini	
tribe	Heresiarchini	
tribe	Ichneumonini	
tribe	Joppocryptini	
tribe	Listrodromini	
tribe	Oedicephalini	
tribe	Phaeogenini	
tribe	Platylabini	
tribe	Zimmerini	
subfamily	Lycorininae	
subfamily	Mesochorinae	
subfamily	Metopiinae	
subfamily	Neorhacodinae	
subfamily	Ophioninae	
subfamily	Orthocentrinae	
subfamily	Orthopelmatinae	
subfamily	Oxytorinae	
subfamily	Phrudinae	
subfamily	Pimplinae	
tribe	Delomeristini	
tribe	Ephialtini	
tribe	Perithoini	
tribe	Pimplini	
tribe	Polysphinctini	
subfamily	Poemeniinae	
tribe	Poemeniini	
tribe	Pseudorhyssini	
subfamily	Rhyssinae	
subfamily	Stilbopinae	
subfamily	Tersilochinae	
subfamily	Tryphoninae	
tribe	Eclytini	
tribe	Exenterini	
tribe	Idiogrammatini	
tribe	Oedemopsini	
tribe	Phytodietini	
tribe	Sphinctini	
tribe	Tryphonini	
subfamily	Xoridinae	
superfamily	Cephoidea	
family	Cephidae	
superfamily	Orussoidea	
family	Orussidae	
superfamily	Pamphilioidea	
family	Megalodontesidae	
family	Pamphiliidae	
subfamily	Pamphiliinae	
subfamily	Cephalciinae	
superfamily	Siricoidea	
family	Siricidae	
subfamily	Siricinae	
subfamily	Tremicinae	
superfamily	Tenthredinoidea	
family	Argidae	
subfamily	Arginae	
subfamily	Sterictiphorinae	
family	Blasticotomidae	
family	Cimbicidae	
subfamily	Abiinae	
subfamily	Cimbicinae (now Corynidinae)	
subfamily	Coryninae	
family	Diprionidae	
subfamily	Diprioninae	
subfamily	Monocteninae	
family	Tenthredinidae	
subfamily	Allantinae	
subfamily	Blennocampinae	
subfamily	Heterarthrinae	
subfamily	Nematinae	
subfamily	Selandriinae	
subfamily	Tenthredininae	
superfamily	Xiphydrioidea	
family	Xiphydriidae	
superfamily	Xyeloidea	
family	Xyelidae	

## Temporal coverage

**Living time period:** Currently living.

### Notes

Currently living animals in stable populations, largely excluding (1) rare / irregular immigrants, intruder or invader species, (2) accidental or deliberate releases of exotic (pet)species, (3) domesticated animals, (4) foreign species imported and released for bio-control or (5) foreign species largely confined to hothouses.

## Usage rights

### Use license

Open Data Commons Attribution License

### IP rights notes

*Fauna Europaea* data are licensed under CC BY SA version 4.0. The experts keep property rights over their data, initially covered under the FaEu-SMEBD conditions. For more copyrights and citation details see: https://fauna-eu.org.

For correct use and citing of the Taxapad data set, please check the relevant website.

## Data resources

### Data package title

Fauna Europaea - Hymenoptera-Symphyta

### Resource link

http://www.faunaeur.org/Data_papers/FaEu_Hymenoptera-Symphyta_2.6.2.zip

### Alternative identifiers

http://www.faunaeur.org/experts.php?id=118

### Number of data sets

2

### Data set 1.

#### Data set name

Fauna Europaea - Hymenoptera-Symphyta version 2.6.2 - species

#### Data format

CSV

#### Number of columns

25

#### Character set

UTF-8

#### Download URL

http://www.faunaeur.org/Data_papers/FaEu_Hymenoptera-Symphyta_2.6.2.zip

#### 

**Data set 1.** 

Column label	Column description
datasetName	The name identifying the data set from which the record was derived (http://rs.tdwg.org/dwc/terms/datasetName).
version	Release version of data set
versionIssued	Issue data of data set version
rights	Information about rights held in and over the resource (http://purl.org/dc/terms/rights)
rightsHolder	A person or organization owning or managing rights over the resource (http://purl.org/dc/terms/rightsHolder)
accessRights	Information about who can access the resource or an indication of its security status (http://purl.org/dc/terms/accessRights)
taxonID	An identifier for the set of taxon information (http://rs.tdwg.org/dwc/terms/taxonID)
parentNameUsageID	An identifier for the name usage of the direct parent taxon (in a classification) of the most specific element of the scientific Name (http://rs.tdwg.org/dwc/terms/parentNameUsageID)
scientificName	The full scientific name, with authorship and date information if known (http://rs.tdwg.org/dwc/terms/scientificName)
acceptedNameUsage	The full name, with authorship and date information if known, of the currently valid (zoological) taxon (http://rs.tdwg.org/dwc/terms/acceptedNameUsage)
originalNameUsage	The original combination (genus and species group names), as firstly established under the rules of the associated nomenclatural Code (http://rs.tdwg.org/dwc/terms/originalNameUsage)
family	The full scientific name of the family in which the taxon is classified (http://rs.tdwg.org/dwc/terms/family)
familyNameId	An identifier for the family name
genus	The full scientific name of the genus in which the taxon is classified (http://rs.tdwg.org/dwc/terms/genus)
subgenus	The full scientific name of the subgenus in which the taxon is classified. Values include the genus to avoid homonym confusion (http://rs.tdwg.org/dwc/terms/subgenus)
specificEpithet	The name of the first or species epithet of the scientific Name (http://rs.tdwg.org/dwc/terms/specificEpithet)
infraspecificEpithet	The name of the lowest or terminal infraspecific epithet of the scientificName, excluding any rank designation (http://rs.tdwg.org/dwc/terms/infraspecificEpithet)
taxonRank	The taxonomic rank of the most specific name in the scientific Name (http://rs.tdwg.org/dwc/terms/infraspecificEpithet)
scientificNameAuthorship	The authorship information for the scientific Name formatted according to the conventions of the applicable nomenclatural Code (http://rs.tdwg.org/dwc/terms/scientificNameAuthorship)
authorName	Author name information
namePublishedInYear	The four-digit year in which the scientificName was published (http://rs.tdwg.org/dwc/terms/namePublishedInYear)
Brackets	Annotation if authorship should be put between parentheses
nomenclaturalCode	The nomenclatural code under which the scientific Name is constructed (http://rs.tdwg.org/dwc/terms/nomenclaturalCode)
taxonomicStatus	The status of the use of the scientificName as a label for a taxon (http://rs.tdwg.org/dwc/terms/taxonomicStatus)
resourceDescription	An account of the resource, including a data-paper DOI (http://purl.org/dc/terms/description)

### Data set 2.

#### Data set name

Fauna Europaea - Hymenoptera-Symphyta 2.6.2 - hierarchy

#### Data format

CSV

#### Number of columns

12

#### Character set

UTF-8

#### Download URL

http://www.faunaeur.org/Data_papers/FaEu_Hymenoptera-Symphyta_2.6.2.zip

#### 

**Data set 2.** 

Column label	Column description
datasetName	The name identifying the data set from which the record was derived (http://rs.tdwg.org/dwc/terms/datasetName)
version	Release version of data set
versionIssued	Issue data of data set version
rights	Information about rights held in and over the resource (http://purl.org/dc/terms/rights)
rightsHolder	A person or organization owning or managing rights over the resource (http://purl.org/dc/terms/rightsHolder)
accessRights	Information about who can access the resource or an indication of its security status (http://purl.org/dc/terms/accessRights)
taxonName	The full scientific name of the higher-level taxon
scientificNameAuthorship	The authorship information for the scientific Name formatted according to the conventions of the applicable nomenclatural Code (http://rs.tdwg.org/dwc/terms/scientificNameAuthorship)
taxonRank	The taxonomic rank of the most specific name in the scientific Name (http://rs.tdwg.org/dwc/terms/infraspecificEpithet)
taxonID	An identifier for the set of taxon information (http://rs.tdwg.org/dwc/terms/taxonID)
parentNameUsageID	An identifier for the name usage of the direct parent taxon (in a classification) of the most specific element of the scientific Name (http://rs.tdwg.org/dwc/terms/parentNameUsageID)
resourceDescription	An account of the resource, including a data-paper DOI (http://purl.org/dc/terms/description)

## Supplementary Material

Supplementary material 1 Ichneumonoidea — Fauna Europaea mapping Data type: xlsx
Brief description: Cross-validation of Fauna Europaea (version 2.6.2) and Taxapad (version 2016). Discrepancies are annotated. For details on data ownership and correct citation please check the Fauna Europaea and Taxapad websites.
File: oo_145108.xlsx Kees van Achterberg, Dicky Sick Ki Yu and Yde de Jong

Supplementary material 2 Fauna Europaea Guidelines for Group Coordinators and Taxonomic Specialists Data type: pdf
File: oo_140930.pdf Yde de Jong, Verner Michelsen, Nicola Bailly

## Figures and Tables

**Table 1.** Responsible specialists per family in Hymenoptera — Symphyta + Ichneumonidae.

**TAXONOMY**		**EUROPE**				
**FAMILY**	**SPECIALIST(S)**	**DATABASED SPECIES (Fauna Europaea)**	**UPDATED NUMBERS (Fauna Europaea)**	**TOTAL DESCRIBED SPECIES (information-gap)**	**TOTAL ESTIMATED SPECIES (knowledge-gap)**	**COMMENT**
Argidae	Andreas Taeger & Stephan Blank	64	55	71	c. 78	
Blasticotomidae	Andreas Taeger & Stephan Blank	1	1	1	1	
Braconidae	Kees van Achterberg	3499	3602	3615	c. 4800	Resource: Taxapad 2012
Cephidae	Andreas Taeger & Stephan Blank	43	43	44	c. 48	
Cimbicidae	Andreas Taeger & Stephan Blank	54	48	49	c. 54	
Diprionidae	Andreas Taeger & Stephan Blank	23	19	19	c. 21	
Heptamelidae (nov. fam.)		0 [3 as Tenthredinidae]	3			
Ichneumonidae	Kees Zwakhals	5865	5865	6644	c. 7300	Resource: Taxapad 2012
Megalodontesidae	Andreas Taeger & Stephan Blank	22	22	25	c. 27	
Orussidae:	Andreas Taeger & Stephan Blank	7	7	6	c. 7	
Pamphiliidae	Andreas Taeger & Stephan Blank	60	55	59		
Siricidae	Andreas Taeger & Stephan Blank	19	17	19	c. 21	
Tenthredinidae	Andreas Taeger & Stephan Blank	1042	1001	1034	c. 1130 [incl. Heptamelidae]	
Xiphydriidae	Andreas Taeger & Stephan Blank	7	7	9	c. 10	
Xyelidae	Andreas Taeger & Stephan Blank	11	15	15	c. 17	

**Table 2.** Responsible associated specialists in Hymenoptera — Symphyta + Ichneumonidae.

**GROUP or AREA**	**SPECIALIST(S)**
Pamphiliidae	Matti Viitasaari
Ichneumonoidea	Dicky Sick Ki Yu (Taxapad)
